# Synthesizing Human Activity for Data Generation

**DOI:** 10.3390/jimaging9100204

**Published:** 2023-09-29

**Authors:** Ana Romero, Pedro Carvalho, Luís Côrte-Real, Américo Pereira

**Affiliations:** 1 Faculdade de Engenharia, Universidade do Porto, 4200-465 Porto, Portugalluis.corte-real@inesctec.pt (L.C.-R.); americo.j.pereira@inesctec.pt (A.P.); 2 Instituto de Engenharia de Sistemas e Computadores, Tecnologia e Ciência, 4200-465 Porto, Portugal; 3 School of Engineering, Polytechnic of Porto, 4200-072 Porto, Portugal

**Keywords:** synthetic humans generation, 3D human body, pose estimation, data augmentation, action recognition, computer vision

## Abstract

The problem of gathering sufficiently representative data, such as those about human actions, shapes, and facial expressions, is costly and time-consuming and also requires training robust models. This has led to the creation of techniques such as transfer learning or data augmentation. However, these are often insufficient. To address this, we propose a semi-automated mechanism that allows the generation and editing of visual scenes with synthetic humans performing various actions, with features such as background modification and manual adjustments of the 3D avatars to allow users to create data with greater variability. We also propose an evaluation methodology for assessing the results obtained using our method, which is two-fold: (i) the usage of an action classifier on the output data resulting from the mechanism and (ii) the generation of masks of the avatars and the actors to compare them through segmentation. The avatars were robust to occlusion, and their actions were recognizable and accurate to their respective input actors. The results also showed that even though the action classifier concentrates on the pose and movement of the synthetic humans, it strongly depends on contextual information to precisely recognize the actions. Generating the avatars for complex activities also proved problematic for action recognition and the clean and precise formation of the masks.

## 1. Introduction

The problem of inferring human activity from images is a long-standing problem in computer vision [1]. Over the last two decades, researchers have tackled this problem via the prediction of 2D content, such as keypoints, silhouettes, and part segmentations [2]. More recently, however, the interest has shifted toward retrieving 3D meshes of human bodies, including facial and hand expressiveness, as a result of developments in statistical body models [3]. Models of human bodies [3,4,5,6] are central to this trend due to their flexibility in accurately representing a wide range of poses without being overly complex. The use of these models of human bodies allows researchers to analyze and recreate intricate human actions.

Human actions, behaviors, and interactions with the environment are highly diverse due to the wide range of poses, motions, and facial expressions that humans are capable of and how subtle changes in them can correspond to a wide range of actions. Therefore, datasets must be substantial in size in order to be sufficiently representative of human actions [7]. However, creating such large datasets is a costly and time-consuming task due to the manual labor involved in labeling data. Techniques such as data augmentation help alleviate this issue by artificially expanding modestly sized datasets into much larger and richer datasets without further manual labeling. Nevertheless, we cannot disregard the significant cost and privacy and legal issues arising from the presence of people. Hence, a potential solution to overcome the issue of insufficient data is through transfer learning [8], which consists of pre-training models on large general-purpose datasets and subsequently fine-tuning them on smaller datasets. By embedding the knowledge and experience of a large-scale dataset in the pre-trained model, we reduce the number of data required for the intended application. Data augmentation is a technique that alleviates the challenge of annotating large numbers of data. It consists of artificially generating new labeled data samples, thus enlarging the original dataset without further need for manual labeling. An example of data augmentation is presented in [9], which introduces an augmentation approach of datasets with annotated synthetic images exhibiting new poses. Another example where data augmentation is applied indirectly, i.e., instead of artificially extending a dataset, is SURREAL [10], which is a fully synthetic dataset. The dataset was created with the Skinned Multi-Person Linear(SMPL) body model and driven by 3D motion captures, where parameters were altered, such as illumination, camera viewpoint, and background, to augment the diversity.

The usage of synthetic data is important in multiple scenarios and applications. For instance, we can use accurate virtual humans for human motion analysis and recognition [11,12] and in other major application areas of 3D mesh reconstruction, such as virtual and augmented reality, to create realistic, life-like avatars for interactive experiences [13]; ergonomic analysis [14]; and video games and animation [15,16]. Furthermore, there is also an application in inserting real humans into virtual environments. A notable example of this is using virtual production [17], where virtual and physical film-making techniques are combined to produce media that include human actors in photo-realistic virtual environments, thus saving time and resources in post-production that would otherwise be required. Nowadays, the usage of synthetic and virtual data is even more prominent, and we can see it in all the television media. Another important aspect of this is the fact that synthetic data can also be a valuable resource to train deep learning models. For instance, in [18], synthetic datasets and real datasets augmented with synthetic humans were used as an experiment to show that mixing synthetic humans with real backgrounds can indeed be used to train pose-estimation networks. Another work in [19] explores the possibility of pre-training an action-recognition network in purely synthetic datasets and then transfer-learning it to real datasets with different categories from the ones on the synthetic datasets, showing competitive results. A survey exploring application domains of synthetic data in human analysis is presented in [20], which further highlights the practical usage of synthetic data in multiple application scenarios.

In this paper, we propose a semi-automated framework to generate dynamic scenes featuring synthetic humans performing diverse actions. The framework contains features encompassing background manipulation, avatar size, and placement adjustments, facilitating the creation of datasets characterized by heightened variability and customization that can aid users in creating mixed-reality or purely virtual datasets for specific human activity tasks. We also propose an evaluation methodology for assessing the resulting synthetic videos, as well as the synthetic human models. Experimental results showed that the action classifier used to assess the framework results primarily relied on determining the pose and kinetics of the avatars to determine a precise action identification. Still, the background and the presence of objects in which the actors interact also affect the recognition of the action. We also observed that the Part Attention REgressor (PARE) model also performs better when activities are less complex and when there is less partial occlusion in input videos.

The remaining sections of this paper are organized as follows: Section 2 describes the related work about 3D human pose estimation, reconstruction of human meshes, and data augmentation, while Section 3 introduces our framework, explaining in detail each component, how the PARE’s method generates the avatars, and the additional features in our platform. Section 4 presents our evaluation methods, as well as their results and interpretation. Lastly, in Section 5, we exhibit our main conclusions.

## 2. Related Work

In this section, we explore works that target different areas related to generating synthetic scenes with 3D humans in them. More specifically, we explore methods for 3D human pose estimation, human mesh recovery, and, more broadly, data augmentation.

### 2.1. Three-Dimensional Human Pose Estimation

Three-dimensional human pose estimation is the process of estimating the position and orientation of a person’s limbs and joints in 3D space from images or video. Three-dimensional pose estimation has found applications in various domains [21,22,23], including motion capture or animation for tracking human motion, analyzing human behavior and posture, and generating virtual avatars for gaming or virtual reality. As a result of the human body’s complexity and the variability in appearance and pose, 3D pose estimation requires robust algorithms and careful calibration to handle challenges such as occlusions.

Human activity encompasses a wide range of intricate dynamics that go beyond static positions. It includes the nuanced interplay of muscle activation, joint torques, and the coordination of multiple body segments, resulting in fluid motions and gestures. So it is important to note that 3D human pose estimationrefers to the orientation and position of a human body in 3D space and not just the flat, 2D representation typically captured by cameras or images. There are several approaches to 3D pose estimation, which can be split into two categories: two-stage and direct estimation. The two-stage methodology consists of the prediction of 2D joint locations using pose detectors or ground truth information, followed by a prediction of 3D joint locations from the 2D locations or model fitting, where a common approach exploits a learned dictionary of 3D poses [21]. Even though these methods are more robust to domain shift, they rely too heavily on 2D joint detection for estimating 3D pose and may discard valuable information about the RGB image. Direct estimation consists of directly estimating the body pose from the data without using a pre-defined model. This can be carried out using techniques such as deep learning or geometry-based methods. In general, direct methods tend to be more accurate than model-based methods, but they also require more data and computational resources.

The process of estimating a human pose is used in multiple highly regarded works such as [21,24,25,26,27,28,29]. Furthermore, for additional work, we suggest the surveys [30,31,32,33] to the reader. As we can see, using 3D information about objects and people has led to numerous research works, which naturally stem from a wide range of practical applications in multiple areas, namely, works on (i) posture tracking in clinical environments in the medical field [34]; (ii) the capture of the real 3D motion of the hand using pose estimation systems in the field of Human–Computer Interaction (HCI ) [35]; (iii) pose estimation as a way to analyze the posture and movements of athletes in the field of sports [36].

We can also predict poses using frameworks or tools in other ways. For instance, action classifiers also help to estimate human poses by providing insights into human actions and movements. One notable framework for action classification is MMAction2 [37], which combines state-of-the-art techniques in video understanding, including temporal modeling, spatial-temporal alignment, and multi-modal fusion. These techniques enable MMAction2 to recognize and classify various human actions effectively, providing valuable input for 3D human pose estimation. Frameworks like Detectron2 [38] also enhance the accuracy and robustness of pose-estimation systems by using robust object detection, Instance Segmentation capabilities, and keypoint detection models.

### 2.2. Reconstruction of Human Meshes

Human mesh reconstruction refers to the process of creating a 3D model of a human body or body parts using a series of 2D images or a 3D scanner. This can be carried out using various techniques and technologies, such as 3D scanning, computer vision algorithms, and depth sensors. Regardless of the method used, human mesh reconstruction typically involves several steps, including the pre-processing of the input data, the alignment of the 2D images or scans, and surface reconstruction to create the final 3D mesh model.

Typically, the reconstruction/recovery of human meshes involves estimating the 3D shape and surface properties of a body, such as the position of the bones, muscles, and skin, as well as the texture and appearance of the skin [39]. Pose estimation, on the other hand, estimates the 3D orientation and position of the body relative to some reference frame [39]. In some cases, human mesh reconstruction and pose estimation may be performed separately, with the results of one process being used as input to the other. For example, a 3D mesh model of a person’s body might be used to guide the estimation of the pose of the person in an image or video [24]. Alternatively, the results of pose estimation might be used to refine or improve a human mesh model [21].

As previously stated, there are two approaches to 3D human mesh reconstruction, namely parametric and non-parametric. Both of these approaches can be categorized by their outputs.

- Clothed Body Mesh: a 3D model of a person that is rigged (meaning it has a skeleton and joints that allow it to move) with some clothing details on the surface of the model.
- Naked Body Mesh: a 3D model of a person’s body without any clothing details, only a smoothed surface. These are often used as a base for creating clothed body meshes, as they allow for the clothing to be added and draped over the body in a realistic way.
- Body Mesh with Motion: an animated naked or clothed body mesh, or an output body model that tracks the body’s motion from input video data. These motion data can include animations that depict the character walking, running, jumping, or performing other actions.

One of the main reasons why human mesh reconstruction is so widely used is that it allows for the creation of highly accurate and detailed 3D models of the human body. This can be particularly useful in medical research, where accurate 3D models can be used to study the human anatomy and assist in the diagnosis and treatment of various conditions or to create digital avatars that can be used in video games, movies, and other interactive experiences [40,41,42].

### 2.3. Body Models

Body models are representations of the human body that are used to analyze images and videos of humans in order to extract information or perform tasks such as human pose estimation and human mesh recovery. Due to their ability to create full bodies or parts of bodies, they are used to represent features and keypoints extracted from visual input data. Hence, every pose-estimation algorithm agrees upon a body model beforehand due to advantageous characteristics, such as simplicity, efficiency, and the ability to be more general and less specific to a particular individual. To enable customization and enhance realism and expressiveness, we must use parameters in body models, since these play a crucial role in accurately representing the shape, pose, and kinematics of the human body. The important parameters commonly used in body models include the following.

- The shape template, which is a baseline representation of the body’s shape in a neutral position. It is used as a reference to obtain variations in the shapes and sizes of the body [4];
- The kinematic tree, which defines the hierarchical structure of the body model by indicating the connectivity between different body parts and joints [21];
- The shape, to control the body’s proportions, height, and weight;
- Pose blend shapes, i.e., deformation patterns, which capture local changes in shape as changes in pose occur. These blend shapes are typically defined based on the linear combinations of pose-rotation matrices, allowing the model of the body to accurately deform based on the desired pose [4,5];
- Blend weights determine the influence of different blend shapes on the final shape of the body model [43,44];
- A joint regressor, which is a mapping function that estimates the locations of joints in the body model based on the shape parameters [4,45];

#### 2.3.1. Skinned Multi-Person Linear (SMPL)

The Skinned Multi-Person Linear [4] model is not only compatible with existing graphics software but is also used to represent, as realistically as possible, a broad of body shape variations due to its high accuracy. To that end, the SMPL model can accurately model various human body shapes, be pose-dependent, and display soft-tissue dynamics and compatibility with existing rendering engines. The main characteristic of this model is its simplicity, which enables learning with large amounts of data, leading to better performance. Good-quality data is crucial, and the parameters of this model are learned from 3D scans of different subjects in a wide variety of poses. The 3D scans also consist of the mean template shape (rest pose), blend weights, pose-dependent blend shapes (a linear function of pose rotation matrices), identity-dependent blend shapes, and a regressor from shape to joint locations (to minimize the vertex error, i.e., the discrepancy between the actual locations of the vertices in a 3D model and their corresponding desired or target positions).

SMPL is usually evaluated in two forms: model generalization and shape generalization. The first type of error evaluates the model’s behavior for new people and poses, based on how well it fits in their meshes, and the second is related to the capacity of forming new poses. Thus, model and test registrations are compared using the mean absolute vertex-to-vertex distances.

The SMPL model was trained on thousands of scans of different people with varying poses and is capable of learning shape parameters from large numbers of data while directly minimizing vertex reconstruction error.

SMPL-X [3] and SMPLify [46] represent notable advancements built upon the foundational SMPL model, each addressing specific human body-modeling and analysis aspects. SMPL-X extends the original framework by incorporating a higher degree of expressiveness, allowing it to encompass a more comprehensive array of body shapes and facial expressions by integrating the Faces Learned with an Articulated Model and Expressions (FLAME) and Model with Articulated and Non-rigid defOrmations (MANO) models. This enhanced model permits more detailed and nuanced animations and can directly regress parameters from images. On the other hand, SMPLify enhances the fitting process of the SMPL model to real-world data. Leveraging optimization techniques, SMPLify focuses on accurately aligning the SMPL model to real-world 2D images. This iterative fitting process involves fine-tuning pose and shape parameters to minimize the disparity between the projected SMPL model and the observed data.

#### 2.3.2. Model with Articulated and Non-Rigid defOrmations (MANO)

While many methods treat the 3D modeling and tracking of bodies and hands separately, the Model with Articulated and Non-rigid defOrmations [5] hand model is focused on the creation of an approach to capture the 4D motion of hands and body together. When it comes to body scanners, it can be very hard to resolve hands, especially fingers, which can result in noise and “webbing” between the fingers, i.e., two or more fingers fused. This leads to the occlusion of the hand and the body, which often results in significant missing data. For this reason, MANO deals with noise and missing data and is combined with a parameterized 3D body model (SMPL). This combination enables natural performance capture even under severely noisy measurements.

As previously explained, the need for more and richer training datasets (for example, using dynamic 4D scan sequences instead of static 3D scans) will continuously be an issue, since they are crucial for improving the accuracy of robust body models. Thus, the database created to train this model consisted of a collection of detailed hand scans from 31 persons doing different poses, in which some interacted with objects. MANO learns from similar parameters to those in SMPL, such as template shape, kinematic tree, shape, pose blend shapes, blend weights, and a joint regressor. With this said, MANO is built as a statistical hand model to minimize the vertex error in the training set, just like the SMPL model.

#### 2.3.3. Faces Learned with an Articulated Model and Expressions (FLAME)

Faces Learned with an Articulated Model and Expressions [6] is a fully articulated head model, which means that it can generate faces with different poses and expressions. The model learns from thousands of accurately aligned 3D facial scans in order to minimize facial-reconstruction errors. FLAME is compatible with existing rendering systems and is easy to fit to data; i.e., it can accurately capture the patterns in the data using just a few parameters.

The 3D face modeling field has a large gap between high-end and low-end methods (terms used to refer to the complexity and computational cost of different techniques of creation or rendering of 3D objects). High-end methods are known for having the best facial animation (more realistic) due to more complex algorithms, extensive manual labor, and specialized equipment, such as high-resolution cameras, depth sensors, and motion capture systems with many markers. On the other hand, low-end methods are simpler and more accessible techniques that focus on capturing basic facial expressions and movements with less complexity and resource requirements. FLAME is able to learn a model of facial shape and expression based on sequences of 3D scans and is more expressive than other models, such as the FaceWarehouse [47] and the Basel Face [48] models. The FaceWarehouse model is a database of 3D scans of facial expression of individuals, and the Basel Face model is a 3D morphable model of faces that captures variations in the shapes and textures of human faces. The three models were compared according to their ability to account for unseen data by fitting them to static and dynamic 3D data that are not part of the training using the same optimization method.

As a result of FLAME’s ability to transfer expressions, one can synthesize new motion sequences by transferring facial expressions from source actors to target actors while preserving the subject-specific details of the target face.

### 2.4. Pose and Human Mesh Estimators

Pose and human mesh estimators are algorithms used to estimate the pose and shape of a human body in images and videos. Overall, these algorithms are important tools for understanding and analyzing the movements and behaviors of humans in images and videos.

#### 2.4.1. Human Mesh Recovery (HMR)

Human mesh recovery [21] is an end-to-end framework that produces a richer and more useful mesh representation of a human body in 3D through a single RGB image. This framework uses the SMPL model to parametrize meshes based on the joint angles and shapes (through a low-dimensional linear space).

Even though most approaches focus on recovering 3D joint locations, the estimation of the full body pose is non-trivial since the location of the joints does not define all the degrees of freedom at each joint [21]. HMR implicitly learns the joint angle limits from datasets of 3D body models. Thus, the framework begins by receiving an image, then infers the 3D mesh parameters, which minimize the joint reprojection error, before projecting the 3D keypoints to match the annotated 2D keypoints.

HMR is intended to overcome challenges such as the lack of large-scale datasets with reliable 3D annotations of images in nature since they do not provide enough information for models to generalize images well in the real world. Moreover, it addresses inherent ambiguities when working with 2D-to-3D mapping, or to be more specific, depth ambiguity, where there are multiple 3D body configurations for the same 2D projections.

#### 2.4.2. Video Inference for Body Pose and Shape Estimation (VIBE)

A body’s motion tells us about its behavior in the world, but previous temporal models of human motion do not capture the complexity of real human motions due to insufficient training data [49]. VIBE (Video Inference for Body Pose and Shape Estimation) [24] is a framework intended to exploit temporal information to more accurately estimate the 3D motion of the body from monocular video, through a temporal neural network and training approach.

The VIBE framework was inspired by the HMR framework, as it learns to estimate sequences of 3D body shape poses from in-the-wild videos such that a discriminator cannot distinguish the estimated motions from the motions in the AMASS dataset [50].

#### 2.4.3. Part Attention REgressor (PARE)

The Part Attention REgressor uses information about the visibility of individual body parts to regress 3D human pose and shape and gain robustness to occlusion by learning from body-part-guided attention masks. In other words, PARE introduces a soft attention mechanism that predicts attention masks guided using body parts; thus, by exploiting information about the visibility of individual body parts, it is possible to predict obstructed parts. This method uses deep convolutional neural networks to solve a difficulty presented in most of these types of models: occlusion. The convolutional neural network (CNN) will take an input image to extract its volumetric features, allowing the verification of the visibility of the body parts, checking if their locations are visible or occluded. The 2D Body Branch learns the attention weights for each body part, where each pixel corresponds to a region in the image and stores its correspondent height and width values. The 3D Body Branch predicts the 3D positions of the body joints (through an SMPL regression) based on the weighted input features. Both branches are followed by a module devoted to training attention maps that highlight the relevant regions of the input image for each of the estimated body joints. These attention maps weigh the input features at each pixel, allowing the network to focus on the most important parts of the image when making its predictions. Therefore, the final feature is exploited for a regression of the parameters of a human body model (such as SMPL) and the camera parameters (3D position and orientation).

PARE’s main contribution is the analysis around the influence of occlusion sensitivity in the global pose. By taking advantage of information about the visibility of individual body parts and information from neighboring body parts to predict occluded parts, this soft attention mechanism can overcome issues such as the reliance on global feature representations, making them sensitive to even small occlusions.

#### 2.4.4. DensePose

DensePose [26] establishes dense relationships between 2D images and the 3D representation of the human body by mapping each pixel in the RGB image to a specific location on the surface of the human body. The method is fully supervised and collects correspondences between the persons appearing in the images from the COCO dataset [51] and a surface of a parametric model of the human body, the SMPL model. Thus, an annotation process that allowed the yield of a new dataset, DensePose-COCO [26], was developed with the help of the obtained ground truth correspondences.

The DensePose architecture starts with the feature extraction of the input image using the ResNet50 FPN (Feature Pyramid Network). This CNN accurately locates and identifies the different parts of the body from the input image. The extracted features are then aligned by the RoIAlign (Region of Interest Align) module, allowing for more accurate and robust object detection and pose estimation. The output of the RoIAlign module feeds a series of auxiliary neural network layers for other tasks, such as keypoint estimation and Instance Segmentation. Overall, the combination of these layers is responsible for predicting the 3D coordinates of each pixel in the input image and mapping them to the surface of the human body; i.e., they allow an accurate estimation of the U,V body coordinates. U,V coordinates correspond to the coordinates of a 2D plane to represent a 3D mesh to further texture the model [2].

#### 2.4.5. PoseBERT

PoseBERT [27] is a transformer-based module for the pose sequence modeling of monocular RGB videos, designed to train without the need for any cumbersome RGB image or frame pose annotations. PoseBERT was intended to exploit several of the motion-capture datasets for training better temporal models. Based on the BERT (Bidirectional Encoder Representations from Transformers) model [52], parameters are trained through a masked modeling task, to learn temporal dynamics, i.e., the poses that change over time as they move or perform an action. This learning leads to the correct interpolation of the pose from the previous frame. PoseBERT extracts the input poses more efficiently, even with frames with missing predictions, and can be trained for both body or hand 3D models due to the use of the SMPL [4] and MANO [5] models, respectively.

### 2.5. Data Augmentation

Human poses contain critical information that describes the interaction between human behavior and the surrounding environment. Therefore, identifying human poses over time is crucial for understanding and generating data. Data labeling becomes a challenge as data collection increases, which can be alleviated through data augmentation, i.e., by synthetically increasing the size and diversity of datasets that would otherwise be insufficient.

The authors of [9] proposed a data augmentation methodology for 3D pose estimation that is divided into four phases. In the first phase, 2D and 3D data are collected, where the 2D data consist of in-the-wild images of humans in various poses where the joints are manually labeled, and the 3D data consist of motion capture data. Then, for each 3D pose, a random image from the dataset is selected for each joint with a matching pose. The random images of each 3D pose are then stitched together as a new image. Finally, this process is repeated multiple times for each 3D pose in order to obtain a larger dataset. Another case of data augmentation is SURREAL [10], which is a large scale dataset with synthetically generated but realistic images of people. The synthetic bodies are created using the SMPL model [4] to later on be deformed and rendered. This results in variations in the actions captured from 3D sequences of human motion capture data. Since SMPL is compatible with Blender [53], the parameters of the created body can be altered and rendered on top of other scenes.

Although the use of synthetic data seems like an appealing solution, it also brings some challenges. These challenges revolve around the plausibility of the actions portrayed in the generated avatars. Another challenge is seamlessly integrating a 3D human model into a chosen background to achieve realism, which can be non-trivial. For such a combination to work, creating images that accurately capture color, texture, context, and shadows while accommodating variations in poses, body structure, clothing, and environmental settings is required.

## 3. Avatar Generation Application

Nowadays, the usage of synthetic data has become prominent in multiple application scenarios, ranging from virtual and augmented reality to training data that can be used successfully to train machine learning models. When looking particularly into human behavior analysis and related topics, it is noticeable how important it is to have sufficiently large and variate datasets so that models can better generalize using these data. With this in mind, we propose a framework for the semi-automatic manipulation and generation of visual scenes with synthetic humans performing actions.

**Figure 1:**
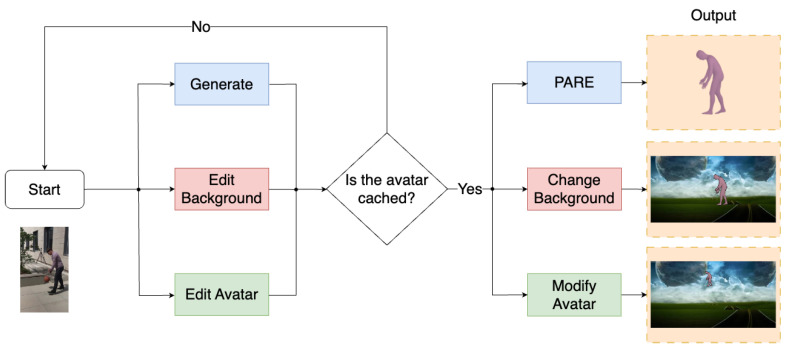
Schematic of the Avatar-Generation Application.

Our framework allows users to select input videos with people performing actions and automatically extracts their pose to generate a virtual avatar performing the same actions. The users can then manipulate these synthetic humans and place them in arbitrary visual or virtual scenes by means of a web application. Figure 1 illustrates the workflow of the designed web application, showing the possible use cases. The process starts by processing an input video that contains a person performing an action and performs human detection, tracking and synthesis, also caching these results. The users can then manipulate the scene and manipulate the synthetic human extracted from the input video.

For synthetic human generation, we chose to use the PARE algorithm, as it uses the well-known Resnet-50 [54] backbone and is partially robust to occlusion. This algorithm starts by first performing human detection and tracking and then rendering each detected human in its original place. This way, the synthetic humans generated by our mechanism replicate the exact actions and positions observed in the input video relative to the captured actor. Internally, the algorithm takes the tracking of each actor and performs a regression of SMPL parameters, which describe the body shape and pose of the synthetic human. Additionally, the PARE model comprises two integral components: learning to regress 3D body parameters and learning attention weights per body part. Firstly, we initialize the frames and the stored bounding boxes to pass them through the PARE model, which yields the predicted camera parameters, 3D vertex coordinates of the SMPL model, predicted pose and shape parameters, and 2D and 3D joint positions. Then, the model learns attention weights for each body part, which serve as a guide, allowing the model to focus on specific regions of interest within the input image. By directing attention to particular body parts, the model can extract more accurate and detailed information, enhancing the overall quality of the generated synthetic humans.

**Figure 2:**
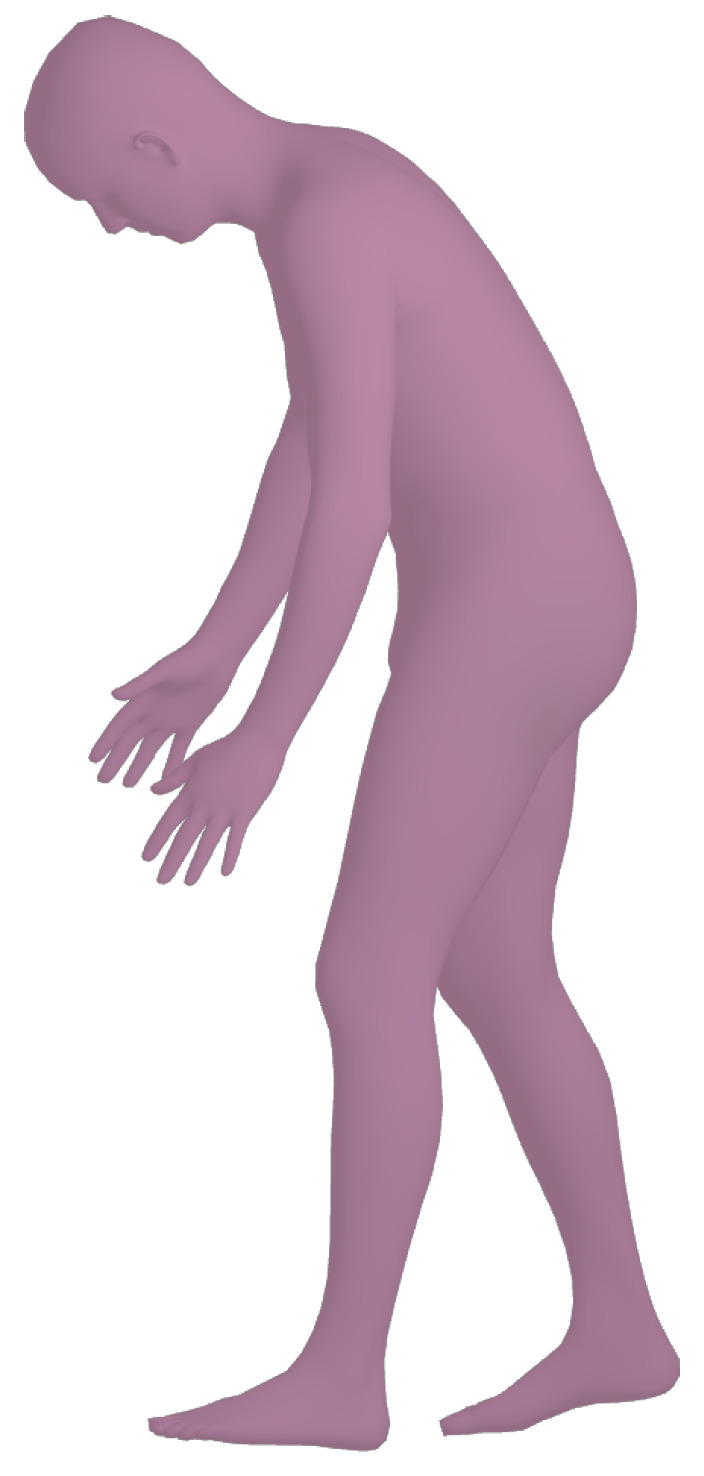
Example of a 3D mesh.

With these data, we are able to render synthetic human bodies like the one depicted in Figure 2. By making use of the predicted camera parameters, shape and pose, we are able to manipulate the resulting avatars and place then on the image plane. We can then make use of our web application to change the size and position of the generated synthetic human, as well as the background where they can be placed. As the algorithm supports the detection of multiple humans, this allows users to even manipulate and generate crowded scenes with synthetic humans.

Overall, our proposal makes use of existing technology and provides a tool that allows users to semi-automatically generate visual data with synthetic humans performing actions based on real scenes. This allows a significant reduction in the manual effort required for data retrieval or collection, which also allows the training and testing of machine learning models in diverse simulated environments that can be crucial in many application scenarios.

## 4. Results

We employed two evaluation methods to assess the quality and usability of the data obtained from our web application: (i) the usage of an action-recognition algorithm on our outputs and (ii) the evaluation of the avatars, in terms of body resemblance, through segmentation. We conducted extensive experiments using publicly available videos, each containing one of the following actions: basketball dribble, archery, boxing, and pushups. The basketball video was from the 3DPW [55] dataset, the archery and boxing videos were from the UCF101 [56] dataset, and the pushups videos were from the HMBD51 [57] dataset (the resulting videos obtained by inserting synthetic humans can be found at https://mct.inesctec.pt/synthesizing-human-activity-for-data-generation, accessed on 26 September 2023).

### 4.1. Action Recognition

For the first experimental phase, we used MMAction2 [37], which is a framework designed for action recognition that gives, as output, the top five labels of its predictions and the respective scores. MMAction2 is known for supporting a comprehensive set of pre-trained models for action recognition and for being flexible and easy to use. Among the several pre-trained action-recognition models available within the framework, we selected the Temporal Segment Networks (TSN) model due to its operability on short video segments to capture spatial and temporal cues. Lastly, all of the frames presented in Table 1 represent the cases in which we tested the action-recognition algorithm.

Table 2 displays the scores for each label given by the MMAction2 that we considered correct, i.e., that corresponded to the action performed by the actors in the original videos and the scores of the avatars substituting the respective actors. It is essential to highlight that the scores presented in the Table are among the top five, not necessarily the top one, score. The MMAction2 was unsuccessful with any cases containing new backgrounds; i.e., it could not predict correctly between the top five action predictions.

We also performed a particular test regarding the basketball-dribbling video, where the ball was not present, due to the unsuccessful attempt to remove the objects which the actor interacted with in the remaining videos. Hence, we observed that in this test case, the action classifier was not able to correctly predict the labels we accepted for this action: dribbling basketball and playing basketball. Thus, the remaining tests concerning this video used the background where the ball appears.

The last stage of this evaluation method consisted of re-adjusting the avatars in terms of placement and size; i.e., for three different sizes for the synthetic humans (original, smaller, and bigger), they were placed more to the left, more to the right, upwards, and downwards. Table 3 exhibits the scores of the action labels given by the MMAction2 (which we considered correct) for the cases of the final part of this evaluation phase, where the cells colored in grey represent cases where the action classifier was unsuccessful in none of its top five predictions. We used the results of the experimental outputs regarding the avatars with the same size and placement as the input actors and on the original background as a reference value. It is visible that the results of the basketball-dribbling and boxing videos were very similar to the respective reference values. Overall, the archery video improved the scores for the avatars with smaller sizes and three other exceptions compared to the avatar with the actual size in the original position. A possible explanation for these results was due to perspective and visual hints; i.e., placing the avatars in different locations and sizes may alter the understatement about what is happening, allowing the model to be more confident about the prediction of the action. Even so, the avatars with a larger size, placed upwards and downwards, showed inferior results but were analogous to the first experiment’s output. Lastly, the classifier could only correctly label two between the twelve cases for the push-up video, namely when we placed the avatar more to the left and upwards, with a bigger size. The explanation for these two deviations could be that they stand out more in the frame due to their size and placement.

### 4.2. Segmentation

The next experimental phase consisted of the evaluation of the segmentation results to evaluate the fit of PARE’s model analytically. We generated masks of the actors and avatars using Detectron2 [38] by employing two models for segmentation already included in the framework: Instance Segmentation and Panoptic Segmentation. For Instance Segmentation, we utilized the Mask R-CNN [58] architecture with the ResNet-50 backbone, while for Panoptic Segmentation, we employed the Panoptic FPN architecture [59] with the ResNet-101 backbone. Table 4 illustrates the segmentation results of the four aforementioned cases.

Afterward, we calculated the IoU metric since it allows us to quantify the accuracy and efficacy of our segmentation process. Table 5 displays the results we obtained for the masks of the four videos.

Table 6 exhibits the positive effect of storing the avatar’s information in the four actions we tested using a GTX 1080 graphics processing unit (GPU).

## 5. Conclusions

In this article, we propose a semi-automated mechanism that generates scenes with synthetic humans performing various actions. To achieve this, we implemented a web application that allows users to select input videos with humans performing actions and automatically extract a 3D model of the person that can be inserted into other videos or backgrounds, where the generation of the synthetic humans was performed by employing the PARE algorithm. The application also allows users to manipulate the 3D model’s position and scale, allowing further customization of the output scene.

We also introduced two evaluation methodologies to assess our proposal. The first assesses the ability of our outputs to be considered for a video with actual humans performing actions. To do so, we employed the MMAction2 framework for videos processed using our proposal and analyzed if the predicted actions were in fact the original actions of the extracted input videos. The results showed that for simple actions, this was achieved. However, it failed in cases where the actions involved interaction with other objects. The second evaluation methodology consisted of assessing the PARE models by comparing segmentation masks. We observed that in complex actions, the resulting segmentation masks could not be correctly used to assess the 3D models. However, for simpler actions, it is evident that this type of assessment can indeed be used. The avatars’ appearance may be visually similar to the background or other objects in the scene, leading to possible confusion for the algorithm and difficulties in accurately segmenting the avatars. This suggests that further research on assessment methodologies for objectively evaluating the quality of 3D-generated models is required.

Lastly, our contribution extends beyond works like SURREAL by providing a unique platform combining personalization, realism, and flexibility. Furthermore, our platform goes beyond the capabilities of SURREAL’s work by empowering users to generate realistic content that reflects their personal preferences and creative vision. We understand that each user has unique requirements and desires when creating virtual content, and our platform embraces this diversity by offering a wide range of customization options. By providing a more personalized approach, we enable users to tailor their generated content to specific scenarios or styles.

## Figures and Tables

**Table 1:** First frame from the output video containing the generated avatar in the input video’s background (first row); the output video containing the generated avatar in a new background (second row); the output video containing the generated avatar in different sizes and positions (third row).

	Action	Basketball Dribble	Archery	Boxing	Push-Up
Video					
Avatar Original Background		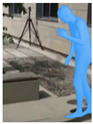	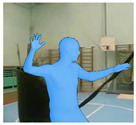	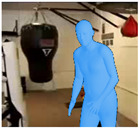	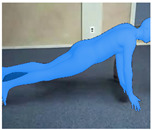
Avatar Different Background		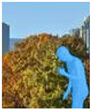	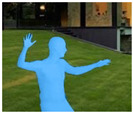	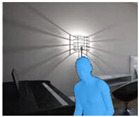	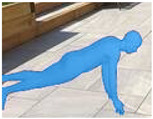
Re-adjusted Avatar		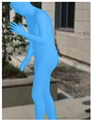	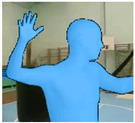	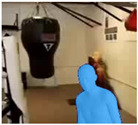	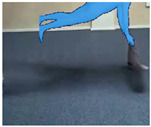

**Table 2:** Scores (in percentages) of the correct labels of the original four videos and of videos containing the avatars in the original background.

Action	Basketball Dribble		Archery	Boxing	Push-Up
Correct label(s)	Dribbling basketball	Playing basketball	Archery	Punching bag	Push-up
Original	66.7	26.7	99.9	99.99	99.9
Avatar (Original Background)	56.7	20.5	19.1	99.97	89.8

**Table 3:** Scores (in percentage) of the correct labels given by the MMAction2 to all the experiments regarding the avatars.

		Basketball Dribble	Archery	Boxing	Push-Up
	**Reference Values**	**56.7**	**19.1**	**99.97**	**89.8**
Original Size	More to the left	50.8	36.9	99.99	-
	More to the right	57.8	5.9	99.996	-
	Upwards	63.5	59.4	99.99	-
	Downwards	50.5	12.3	99.99	-
Smaller Size	More to the left	46.1	40.7	99.99	-
	More to the right	60.4	43.5	99.6	-
	Upwards	67.5	73.8	99.99	-
	Downwards	56.5	42.4	99.99	-
Bigger Size	More to the left	56.0	35.4	99.98	2.3
	More to the right	22.0	-	99.9	-
	Upwards	39.9	8.9	99.998	1.3
	Downwards	26.6	9.2	99.9	-

**Table 4:** Generated mask of the actor and avatar in the first frame from the original video (first row) and from the output video containing the generated avatar in the input video’s background (second row) using Detectron2.

	Action	Basketball Dribble	Archery	Boxing	Push-Up
Video					
Original Instant Segmentation		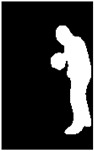	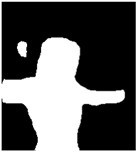	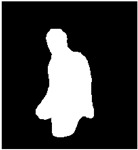	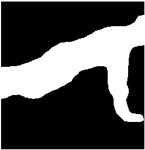
Avatar Instant Segmentation		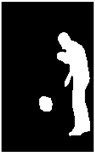	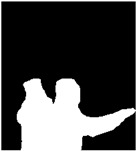	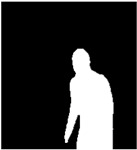	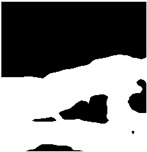
Original Panoptic Segmentation		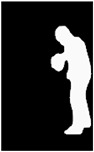	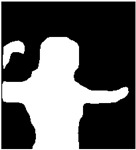	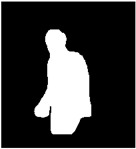	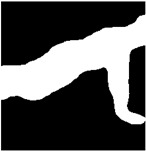
Avatar Panoptic Segmentation		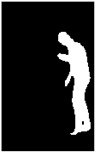	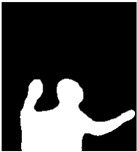	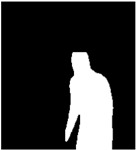	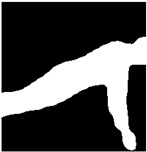

**Table 5:** IoU results using the Instance and Panoptic Segmentation.

	Basketball Dribble	Archery	Boxing	Push-Up
Instance Segmentation (%)	58.53	12.08	47.88	4.28
Panoptic Segmentation (%)	61.86	16.40	47.09	14.69

**Table 6:** Process time for the avatar generation, before and after the avatars are cached, using a GTX 1080 GPU.

	Basketball Dribble	Archery	Boxing	Push-Up
Before caching avatars (seconds)	39.85	31.67	42.39	35.99
After caching avatars (seconds)	17.26	6.77	8.59	8.02
Improvement percentage (%)	56.7	78.6	79.7	77.7

## Data Availability

The resulting dataset obtained by inserting synthetic humans into other backgrounds can be found at the public link https://mct.inesctec.pt/synthesizing-human-activity-for-data-generation (accessed on 26 September 2023).
